# Excessive walking exercise precipitates diabetic neuropathic foot pain: hind paw suspension treadmill exercise experiment in a rat model

**DOI:** 10.1038/s41598-020-67601-6

**Published:** 2020-06-26

**Authors:** Jong Geol Do, Sun Up Noh, Seoung Wan Chae, Kyung Jae Yoon, Yong-Taek Lee

**Affiliations:** 1 Department of Physical and Rehabilitation Medicine, Samsung Medical Center, Sungkyunkwan University School of Medicine, Seoul, Republic of Korea; 2 Department of Physical and Rehabilitation Medicine, Kangbuk Samsung Hospital, Sungkyunkwan University School of Medicine, 29 Saemunan-ro, Jongno-gu, Seoul, 03181 Republic of Korea; 3 Medical Research Institute, Kangbuk Samsung Hospital, Sungkyunkwan University School of Medicine, Seoul, Republic of Korea; 4 Department of Pathology, Kangbuk Samsung Hospital, Sungkyunkwan University School of Medicine, Seoul, Republic of Korea

**Keywords:** Endocrinology, Medical research, Molecular medicine, Neurology

## Abstract

The harmful effects of excessive mechanical loading on diabetic neuropathy and the reason diabetic neuropathic symptoms are common in feet are unclear. In this study, the hind paw suspension treadmill exercise model was used in rats to investigate whether mechanical loading applied to the front paws precipitates neuropathic pain, especially in diabetic conditions. Thirty-two rats were divided into six groups according to the presence of diabetes (DM) and the intensity of mechanical loading applied to the front paws: DM-Hi (high-intensity); DM-Lo (low-intensity); DM-No (non-mechanical loading); Sham-Hi; Sham-Lo; and Sham-No. DM was induced by streptozotocin injection. For high-intensity or low-intensity mechanical loading, treadmill walking exercise was conducted with or without hind paw suspension, respectively. The mechanical withdrawal threshold of the front paw decreased significantly after 8 weeks only in the DM mechanical loading groups (DM-Hi and DM-Lo), and high-intensity loading more significantly decreased the front-paw withdrawal threshold than low-intensity loading. In the DM-Hi group only, macrophage migration inhibitory factor (MIF) increased significantly, and intra-epidermal nerve fibers (IENF) in the front paws decreased significantly. In diabetic conditions, mechanical overloading such as excessive walking is likely to precipitate mechanical allodynia and damage IENF¸ which could explain why diabetic neuropathic symptoms are common in feet. This finding might be related to up-regulation of intracellular signaling cascades such as MIF, rather than inflammatory processes.

## Introduction

Peripheral neuropathy is a common complication of diabetes^1^. The prevalence of diabetic neuropathy is approximately 50% in patients with longstanding disease^2,3^. Hyperglycemia is a key factor underlying diabetic neuropathy because it leads to the accumulation of advanced glycation end products (AGEs), which damages intra-epidermal nerve fibers (IENF) and provokes pain^4–6^.

In diabetic condition, aerobic exercise such as walking is commonly recommended to improve glucose control and reduce microvascular and macrovascular complications^7,8^. Walking exercise can decrease both hyperglycemia-induced damage to nerve cells and neuronal ischemia caused by impaired neurovascular flow in diabetes^9,10^. However, the effects of walking exercise on diabetic neuropathic symptoms are known to vary according to intensity^11^. Moreover, in diabetes, microvascular reactivity in response to extrinsic mechanical stress is impaired, and land-based endurance exercise can harm peripheral nerves^9^. Therefore, it is clinically important to understand the effect of mechanical loading on the development of diabetic neuropathic symptoms.

Diabetic neuropathic symptoms appear in the distal portions of the extremities, most commonly beginning in the feet before the fingers become involved^3,12^. A length-dependent dying back process and fiber-dependent axonopathy (longer fibers are more vulnerable to injury) might be related to this phenomenon^12^. Diabetic nerves also have increased susceptibility to extrinsic stress and are more vulnerable to ischemic damage than non-diabetic nerves^13,14^. However, why diabetic neuropathy occurs most commonly in the feet remains unclear. We hypothesized that mechanical overloading on feet during excessive walking could precipitate neuropathic foot pain, especially in diabetic conditions.

To test that hypothesis, we used the hind paw suspension treadmill exercise in diabetic rats to investigate whether mechanical loading applied to the front paws precipitates neuropathic pain.

## Results

### Assessment of the diabetic rat model

**Figure 1:**
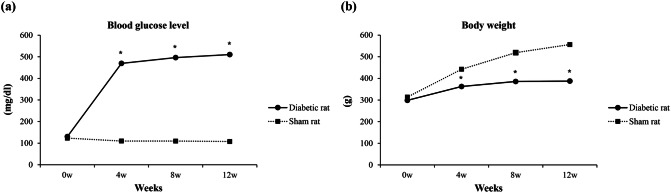
Blood glucose and body weight changes in the rats. (**a**) Blood glucose level and (**b**) body weight changes after streptozotocin (STZ) or saline injection in Sprague Dawley rats. Experimental diabetes was induced by a single intraperitoneal injection of 70 mg/kg STZ to achieve a maximal induced diabetic ratio and maintain a stable and chronic hyperglycemic state. Diabetes was confirmed as a persistent blood glucose level greater than 270 mg/dL. Four weeks after STZ injection, the level of blood glucose in the STZ-injection rats increased significantly compared with the sham rats (*P* < 0.05). The body weight of the STZ-injection rats decreased significantly compared to that of the sham rats (*P* < 0.05). Data are presented as mean ± standard deviation (two-way, repeated measures analysis of variance followed by the post hoc Bonferroni test). ^*^P < 0.05.

To identify the induction of diabetes, we measured blood glucose levels and body weight weekly. The blood glucose levels of the rats were remained below 270 mg/dL in the sham group. The blood glucose levels of rats given a streptozotocin (STZ) injection increased to more than 270 mg/dL by 4 weeks post-injection, which was sustained until 12 weeks (Fig. 1a). The body weights of the diabetic rats decreased significantly compared with the sham rats 4 weeks after STZ-injection (Fig. 1b).

### Time course of mechanical allodynia in the front paws

**Figure 2:**
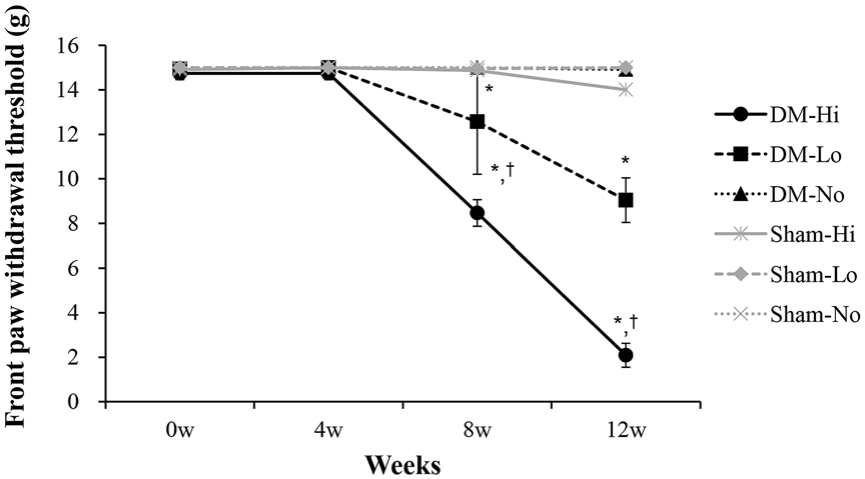
Time course of mechanical allodynia in the front paws. The mechanical withdrawal threshold of the front paws decreased significantly from 8 weeks in the DM mechanical loading groups (DM-Hi and DM-Lo) (*P* < 0.001) but not in the DM-No and sham groups. In the comparison between the DM mechanical loading groups (DM-Hi and DM-Lo), high-intensity mechanical loading decreased the withdrawal threshold for the front paws more significantly than low-intensity loading from 8 to 12 weeks (*P* < 0.001). Data are presented as mean ± standard deviation (two-way, repeated measures analysis of variance followed by the post hoc Bonferroni test). *DM-Hi and DM-Lo vs. Others (*P* < 0.001), ^†^DM-Hi vs. DM-Lo (*P* < 0.001). *DM* diabetes, *DM-Hi* diabetes with high-intensity mechanical loading, *DM-Lo* diabetes with low-intensity mechanical loading, *DM-No* diabetes with non-mechanical loading, *Sham-Hi* non-diabetes with high-intensity mechanical loading, *Sham-Lo* non-diabetes with low-intensity mechanical loading, *Sham-No* non-diabetes with non-mechanical loading.

We investigated the effect of mechanical overloading on mechanical allodynia in the front paws using the hind paw suspension treadmill exercise rat model. Mechanical allodynia was assessed weekly before and after the experiment using the mechanical withdrawal threshold test. The mechanical withdrawal threshold did not differ significantly among the groups at 4 weeks. From 8 weeks, the mechanical withdrawal threshold was decreased significantly in the diabetes (DM) mechanical loading groups (DM-Hi and DM-Lo) but not in the DM-No and sham groups (*P* < 0.001). At 8 weeks, the mechanical withdrawal threshold was 8.5 ± 0.6 g in the DM-Hi group and 12.6 ± 2.4 g in the DM-Lo group. In the comparison between DM-Hi group and DM-Lo group, the high-intensity mechanical loading more significantly decreased the withdrawal threshold of front paw compared to the low-intensity loading after 8–12 weeks (*P* < 0.001). At 12 weeks, the mechanical withdrawal threshold in the DM-Hi group (2.1 ± 0.5 g) was more than fourfold lower than that in the DM-Lo group (9.0 ± 1.0 g) (*P* < 0.001) (Fig. 2). We found that mechanical loading decreased the mechanical withdrawal threshold only under diabetic conditions, and that decrease became more apparent with high intensity and over time.

### mRNA expression levels in the footpad skin of the front paws

**Figure 3:**
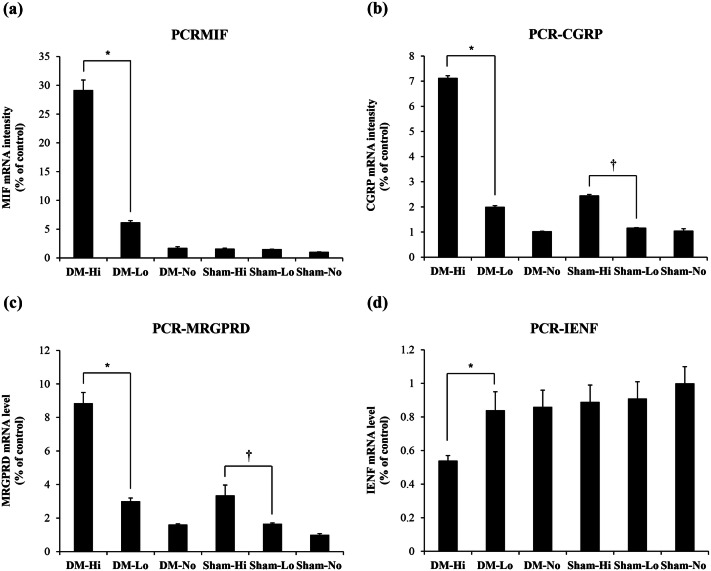
The mRNA expression levels in the footpad skin of the front paws. (**a**) MIF mRNA intensity (%); (**b**) CGRP mRNA intensity (%); (**c**) MRGPRD mRNA intensity (%); (**d**) IENF mRNA intensity (%). The mRNA expression level of MIF increased significantly with high-intensity mechanical loading (*P* < 0.001) only in the DM group, whereas the mRNA expression levels of CGRP and MRGPRD increased significantly irrespective of DM (*P* < 0.001). The mRNA expression level of IENF was significantly lower in the DM- Hi group than in the other groups (*P* < 0.001). *DM-Hi vs*.* DM-Lo (*P* < 0.001), ^†^Sham-Hi vs. Sham-Lo (*P* < 0.001). *MIF *macrophage migration inhibitory factor, *CGRP* calcitonin gene-related peptide, *MRGPRD* Mas-related G protein-coupled receptor D, *IENF* intraepidermal nerve fibers.

After the 12-week experiment, we tested the mRNA expression levels of intracellular signaling cytokines. The macrophage migration inhibitory factor (MIF) increased significantly in the DM-Hi group only (*P* < 0.001), which means high-intensity mechanical loading increases MIF significantly in diabetic condition only. On the other hand, calcitonin gene-related peptide (CGRP) and Mas-related G protein-coupled receptor D (MRGPRD) were increased significantly in both the high-intensity mechanical loading groups irrespective of DM status (*P* < 0.001) (Fig. 3). The mRNA expression of IENF decreased significantly only in the DM-Hi group (*P* < 0.001). The mRNA level of the inflammatory cytokines TNF-α and IL-6 was low in all groups (data not shown).

### Protein expression levels in the footpad skin of the front paws

**Figure 4:**
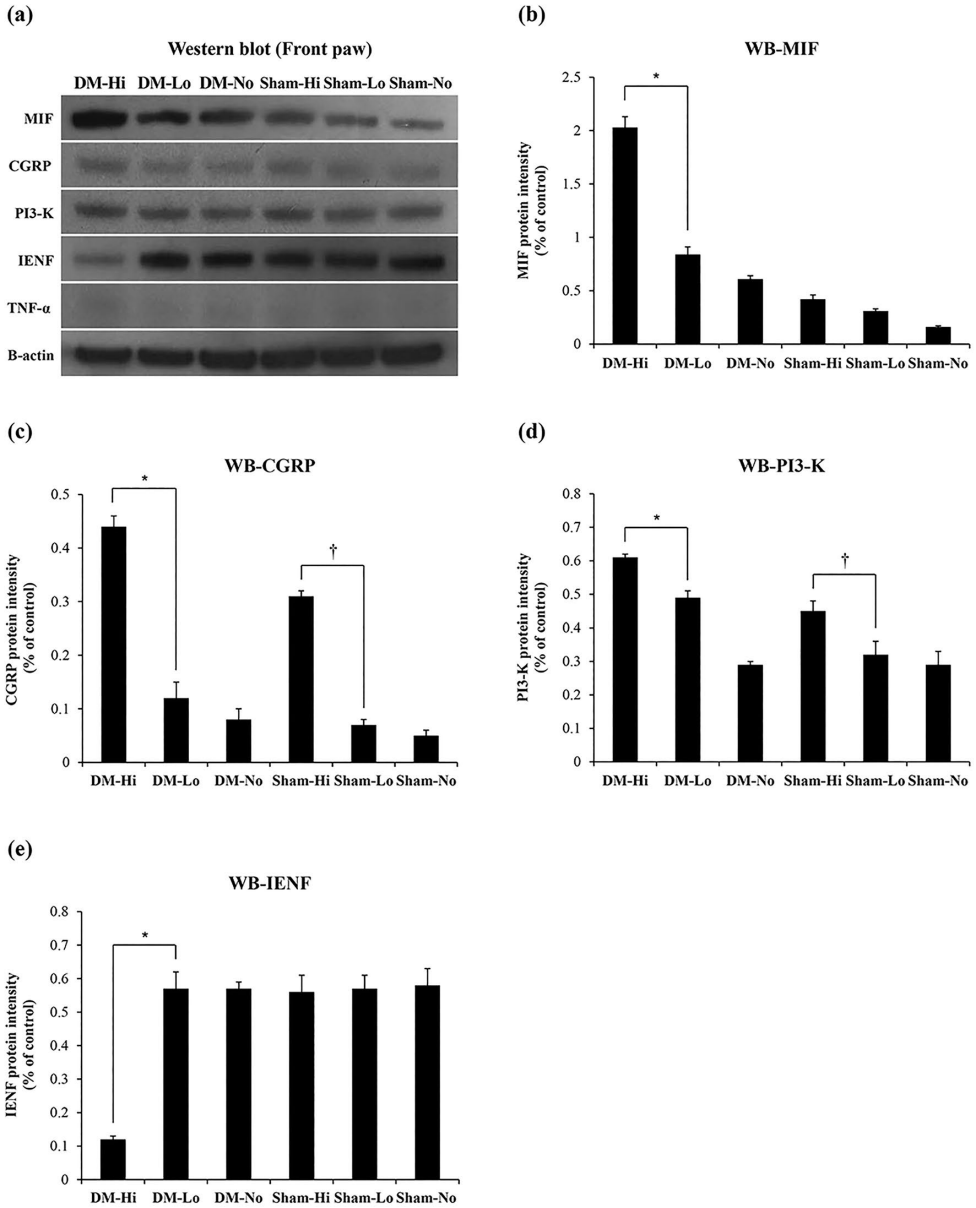
The protein expression levels in the footpad skin of the front paws. (**a**) Western blot of front paw; (**b**) MIF protein intensity (%); (**c**) CGRP protein intensity (%); (**d**) PI3-K protein intensity (%); (**e**) IENF protein intensity (%). The MIF protein expression level increased significantly with high-intensity mechanical loading (*P* < 0.001) only in the DM group, whereas the CGRP and PI3-K protein expression levels increased significantly irrespective of DM (*P* < 0.001). The IENF protein level was significantly lower in the DM-Hi group than in the other groups (*P* < 0.001). *DM-Hi vs. DM-Lo (*P* < 0.001), ^†^Sham-Hi vs*.* Sham-Lo (*P* < 0.001). *DM *diabetes, *MIF* macrophage migration inhibitory factor, *CGRP* calcitonin gene-related peptide, *PI3-K* phosphoinositide 3-kinase, *IENF* intraepidermal nerve fibers, *TNF-α* tumor necrosis factor-alpha.

Western blots showed that high-intensity mechanical loading significantly increased the protein levels of MIF only under diabetic conditions, whereas it markedly increased CGRP and phosphoinositide 3-kinase (PI3-K) irrespective of DM status (Fig. 4). In the DM groups, the protein expression levels of MIF, CGRP, and PI3-K increased as the mechanical loading increased (*P* < 0.001). In the sham groups, the protein expression level of MIF was not elevated, but CGRP and PI3-K did increase as mechanical loading increased (*P* < 0.001). The protein expression level of IENF decreased significantly only in the DM-Hi group (*P* < 0.001). The protein expression level of the inflammatory cytokine TNF-α was low in all groups. These results reveal that mechanical overloading induces the over-expression of the intracellular signaling regulator MIF in the footpad skin of the front paws only in diabetic condition.

## Discussion

The results of this study demonstrate that under diabetic conditions, mechanical allodynia of the front paws increased as the mechanical loading applied to the front paws increased. In addition, high-intensity mechanical loading increased the protein expression of the intracellular signaling regulator MIF and decreased IENF only under diabetic conditions.

The most common manifestation of diabetic neuropathy is distal symmetric polyneuropathy combined with impairment of nerve regeneration and the wide involvement of both large and small nerve fibers. Because of a length-dependent dying back process, the symptoms of diabetic polyneuropathy, such as numbness, tingling, pain, or weakness, begin in the feet before the fingers are involved^4,15^. Diabetic polyneuropathy could be related to the length of a nerve’s axon, so it is initiated in the toes and progresses upward to the calf^12^. Many factors affect the development of diabetic neuropathy. Chronic hyperglycemia can lead to cellular damage in several ways, thereby damaging IENF and provoking pain^12^. Excessive glycolysis generates an overload of reactive oxygen species and reduces nicotinamide adenine dinucleotide phosphate levels, which can lead to oxidative stress. The activation of NF-kB, up-regulation of AGEs receptors, and increased flux through the hexosamine pathway are associated with the inflammatory response^3,4,12^. However, those cellular mechanisms are not enough to explain why diabetic neuropathic symptoms are most common in the feet. Previous studies that evaluated the effects of walking exercise on diabetic neuropathic symptoms using various intensities and durations of exercise found that moderate exercise could suppress the release of numerous neurotransmitters. Belotto et al. reported that moderate treadmill exercise produced marked anti-inflammatory effects in a diabetic rat model^16^. Pitcher, et al. reported that modest amounts of exercise can produce analgesic effects and effectively reduce hypersensitivity and foot stress^17^. However, other studies have reported that exercise has negative effects on diabetic peripheral neuropathy. In an animal study, land-based endurance exercise increased Schwann cell apoptosis in diabetic peripheral nerves^9^. In diabetes, impaired microvascular reactivity in response to extrinsic mechanical stress has been reported^9,18^. Excessive loading and repetitive overloading could cause a loss of protective mechanisms and subsequently lead to neural damage. Moreover, impaired sensory perception and weakened muscles make diabetic nerves particularly vulnerable to mechanical damage. We found that mechanical allodynia increased after a high-intensity mechanical loading exercise in a diabetic rat model, which suggests that excessive walking could have a harmful effect on diabetic neuropathic symptoms. This could explain why diabetic neuropathic symptoms are most common in the feet. Therefore, avoiding mechanical overloading, such as excessive walking, might help to prevent or alleviate diabetic neuropathic pain.

CGRP, MRGPRD, PI3-K, and MIF are known to be involved in nociceptive processing and neuropathic pain^19–21^. CGRP plays an important role in chronic pain by facilitating the introduction of synaptic pain information and maintaining allodynia at the spinal cord level^20^. PI3-K participates in nociception within the spinal cord, dorsal root ganglion, and peripheral nerves. A blockade of spinal PI3-K attenuates injury-induced hypersensitivity^19^. MIF elicits pro-inflammatory signaling in the microglia in the spinal cord, and triggers functional and anatomical plasticity in sensory neurons. In our study, however, mechanical allodynia and MIF expression in front paw increased significantly with high-intensity mechanical loading under diabetic conditions only, whereas the expression of CGRP, MRGPRD and PI3-K increased irrespective of the diabetic condition. In addition, IENF expression in fat pad of front paw decreased significantly with high-intensity mechanical loading under diabetic conditions only. Our results suggest that in diabetic condition, excessive mechanical loading applied to the front paw has a negative-nociceptive effect in foot pad of front paw that is related to the activation of MIF, rather than the CGRP, MRGPRD or PI3-K. In terms of the effect of MIF on neuropathic pain, it has been reported that MIF plays a critical role in inflammatory and neuropathic pain and enhances pain in response to stress^10,22^. In an animal study, mechanical hypersensitivity occurred when recombinant MIF injected into the hind paws of mice, and mechanical hypersensitivity did not occur in MIF knockout mice. In diabetic condition, MIF could aggravate diabetic neuropathy by suppressing glyoxalase-1 (GLO-1)^23^. GLO-1 is known to suppress the accumulation of AGEs by inhibiting glycation, thereby preventing glycation-mediated cell damage^24^. The suppressed GLO-1 is associated with neuronal extracellular matrix protein’s damage^6^. In the previous report, we showed that under diabetic conditions, MIF could aggravate diabetic neuropathic pain, and MIF over-expression was accompanied by low expression of GLO-1 and IENF in the foot pad of hind paw. In this report, MIF gene knockdown keratinocyte with siRNA showed significantly up-regulated IENF expression in hyperglycemic conditions compared with control keratinocytes, which indicate that in hyperglycemic condition, blockade of MIF could protect IENF and decrease diabetic neuropathic pain^23^. These results imply that mechanical stress induces MIF over-expression, especially in diabetic conditions, and that activated MIF might play a role in the development of diabetic neuropathic pain. Therefore, control of mechanical loading or the regulation of MIF might be potential therapeutic targets to alleviate diabetic neuropathic symptoms. However, further investigation is required in order to better dissect the mechanisms involved in the effects of excessive walking exercise and MIF in diabetic neuropathy.

Together, corticosteroids and MIF act as part of a homeostasis to control inflammation and regulate cell stress hormones. MIF is a unique cytokine produced in parallel with corticosteroids in stress conditions, and it can counter-regulate the effects of corticosteroids^24–26^. MIF can overcome the inhibitory effects of glucocorticoids on TNF-α, IL-1, IL-6, and IL-8 and suppress the protective effects of steroids against the acute stress response^27^. Alterations in corticosteroids, MIF, and their downstream signaling mechanisms are related with chronic neurologic dysfunction^28^. Despite the evidence that glucocorticoids and MIF are associated with neuropathic pain, the role of MIF–glucocorticoid interactions in diabetic neuropathy remains poorly defined. Theoretically, mechanical overloading induces cascades of tissue injury, such as inflammation and glucocorticoid release, which activate the release of MIF. Consequently, the role of MIF in inflammation and neuropathic pain and its ability to override the effects of glucocorticoids could cause neuropathic symptoms. To understand the role of MIF–glucocorticoid interactions in diabetic neuropathy during mechanical overloading, the measurement of circulating glucocorticoid levels might be needed.

Several studies have indicated that inflammatory cytokines such as TNF-α, IL-1β, and cytokine-induced neutrophils are associated with endothelial cell dysfunction in diabetes^29–31^. The over-expression of pro-inflammatory cytokines such as TNF-α, IL-2 receptor, IL-1, and C-peptide in diabetic neuropathy suggests that inflammation is associated with diabetic neuropathy^32,33^. Physical exercise can stimulate the immune mechanism, producing an anti-inflammatory cellular process^34^. In an animal study, moderate exercise in the form of running attenuated the expression of the pro-inflammatory cytokines TNF-α and IL-1β in the dorsal root ganglion in diabetic conditions^33^. Also, moderate-intensity treadmill exercise decreased the serum level of TNF-α^35^. The anti-inflammatory effects of exercise training could explain the low protein expression levels of inflammatory cytokines found in our study.

Usually, diabetic neuropathic symptoms occur in parallel with the degeneration of peripheral nerves^36^. Diabetic polyneuropathy increases the risk of foot ulceration up to sevenfold and is a significant risk factor in more than 60% of amputations of the lower limbs. Diabetic neuropathy is a major cause of disability and reduced quality-of-life and the leading cause of non-traumatic limb amputation^1,36,37^. The early detection and assessment of the severity of neuropathy could prevent complications and improve quality-of-life. The assessment of IENF is a useful tool to identify the presence and severity of neuropathy. A reduction in IENF density was prominently observed in patients with diabetic neuropathy, and IENF density is inversely associated with both cold and heat detection thresholds^38, 39^. In a previous study, the presence of neuropathy was significantly associated with a decreased density of IENF in diabetic patients^40^. In our study, IENF expression in the front paws of the DM-Hi group decreased significantly compared with the other groups. This finding suggests that under diabetic conditions, excessive mechanical loading precipitates structural changes, such as a loss of sensory nerve fibers, in the foot pad skin.

There are methodological concerns to conduct further investigation of the upstream pathways and the effect of MIF to better explain the mechanisms associated with the effects of excessive walking on diabetic neuropathy. Mechanical overloading in diabetic condition could induce the activation of nociceptors and trigger an increase in the excitability of the upstream pathway, such as dorsal root ganglion and the dorsal horn of the spinal cord. That effect could lead to spinal disinhibition of nociceptive responses, which is called ‘central sensitization’^41^. Many biological process that can contribute to ‘central sensitization’ have been studied and revealed^42^. However, we did not analyze biochemical changes in the dorsal root ganglion or the dorsal horn of the spinal cord because investigation of ‘central sensitization’ mechanism by mechanical overloading especially in diabetic condition is beyond the scope of this study. The primary purpose of our study was to investigate whether primary nociceptors in foot pad of front paw become more injured and more sensitized in the presence of mechanical overloading especially in diabetic condition, which is called ‘peripheral sensitization’^43^. Nevertheless, further investigation on the mechanisms involved in the effects that excessive walking has on ‘central sensitization’ in diabetic neuropathy would better explain the mechanisms related to diabetic neuropathic pain. Previous studies with diabetic rat model evaluated biochemical changes in lumbar 4–5 level of spinal cord and dorsal root ganglion for the evaluation of upstream pathway of hind paw pain^44^. However, because our study is first to evaluate the front paw for the diabetic neuropathic pain, it is necessary to determine which cervical spine level of the dorsal root ganglion and dorsal horn of spinal cord should be evaluated for the upstream pathway of front paw pain.

In addition, experiment with MIF gene knockout mice or MIF receptor blocker injection would be helpful for the investigation of the effect of MIF and mechanical loading on neuropathic pain in diabetic condition. However, the method for mechanical overloading condition for in vitro study has not been established. For in vivo study, hind-paw suspension exercise in diabetic animal model with MIF gene knockout or receptor blocker injection also has not been established. For example, it has not been studied how much intensity should be applied to front paw in diabetic MIF-knockout mouse model with hind paw suspension exercise, and which spinal level should be selected to perform intrathecal MIF receptor blocker injection to evaluate front paw pain. In addition, MIF blocker injection in foot pad of front paw appeared to be inappropriate for the evaluation of front paw pain after application of excessive mechanical loading to front paw. First of all, an appropriate experimental method should be established to get the proper outcomes. Therefore, further study on the mechanisms involved in the effects that excessive walking has on ‘central sensitization’ in diabetic neuropathy and further experiment with the MIF receptor blocker would be inevitably our next research project.

This study has several limitations. First, all animal studies have inherent limitations. The mechanical sensitivity of responses to external stress may differ between bipedal and quadrupedal animals. Nevertheless, we induced mechanical loading that can mimic excessive walking conditions in humans using the hind paw suspension exercise rat model to apply higher intensity mechanical loading to the front paws than conventional treadmill exercise. In this manner, our study has provided information that could be translated from this animal study to the human condition. Second, we should also have performed a histologic analysis to evaluate the presence of MIF or loss of sensory nerve fibers in the front paws and revealed an anatomical view of the tissue. In our previous study, the PCR and Western blot results showed significantly increased MIF and decreased IENF expression levels in the foot pad of rats with diabetic neuropathy, wherein the results of immunohistochemistry staining showed strongly stained MIF and faintly stained IENF especially in the epidermal layer compared with the sham control rats^24^. Thus, in this study, we could assume the immunohistochemistry staining may show similar findings to the previous study on the basis of the PCR and Western blot findings of the study. However, further histologic analysis of MIF and IENF is still needed to better understand the correlation between the biochemical and functional findings. Third, treadmill exercise with hind paw suspension for 3 h/day can be stressful for rats and might have produced endocrine or other physiological effects that could affect diabetic neuropathic pain. We tried to reduce stress and dehydration by making food and water available ad libitum and keeping the circumstances as comfortable as possible for the rats.

The present study suggests that high-intensity mechanical loading, such as excessive walking, can precipitate mechanical allodynia and damage sensory nerve fibers, especially under diabetic conditions, which could explain why diabetic neuropathic symptoms are most common in feet. The up-regulation of MIF might play a role in the development of diabetic neuropathic symptoms in mechanical overloading condition. Therefore, avoiding excessive walking could be important in preventing and alleviating diabetic neuropathic foot pain.

## Methods

### Animals

Thirty-two male Sprague–Dawley rats (120–150 g; ORIENT BIO, Korea) were used. All experimental procedures were performed in accordance with a protocol approved by the Ethics Committee for Animal Experiments at our institute (approval number: 16-082-D3-N). All rats were housed in a room in the animal center under a 12-h light–dark cycle at a constant temperature (22 °C ± 1 °C). The rats were placed individually in separate cages with food and water ad libitum in a specific pathogen-free environment. All studies were conducted according to ethical guidelines of the International Association for the Study of Pain. No rats died or showed evidence of illness during treatment.

### Induction of diabetes

The rats were randomly divided into a diabetic group and sham group. In the diabetic group, diabetes was induced by a single intraperitoneal injection of 70 mg/kg of STZ (Sigma-Aldrich, St. Louis, MO, USA), which destroys pancreatic cells and causes insulin deficiency. In the sham group, rats were received intraperitoneal phosphate-buffered saline injections. Confirmation of diabetes took place 7 days after STZ injection. Animals with blood glucose level > 270 mg/dL were considered as diabetes. The blood glucose level and body weight were checked every week for 12 weeks after STZ injection. Blood samples from the tail veins were tested with a glucose meter (ACCU-CHEK, Roche, Nutley, NJ, USA).

### Experimental animal groups

The 32 rats were divided into 6 groups according to the presence of DM and the intensity of mechanical loading applied to their front paws: DM-Hi (diabetes with high-intensity mechanical loading, *n* = 6); DM-Lo (diabetes with low-intensity mechanical loading, *n* = 4); DM-No (diabetes with non-mechanical loading, *n* = 6); Sham-Hi (non-diabetes with high-intensity mechanical loading, *n* = 6); Sham-Lo (non-diabetes with low-intensity mechanical loading, *n* = 4); and Sham-No (non-diabetes with non-mechanical loading, *n* = 6).

### Mechanical loading exercise protocol

**Figure 5:**
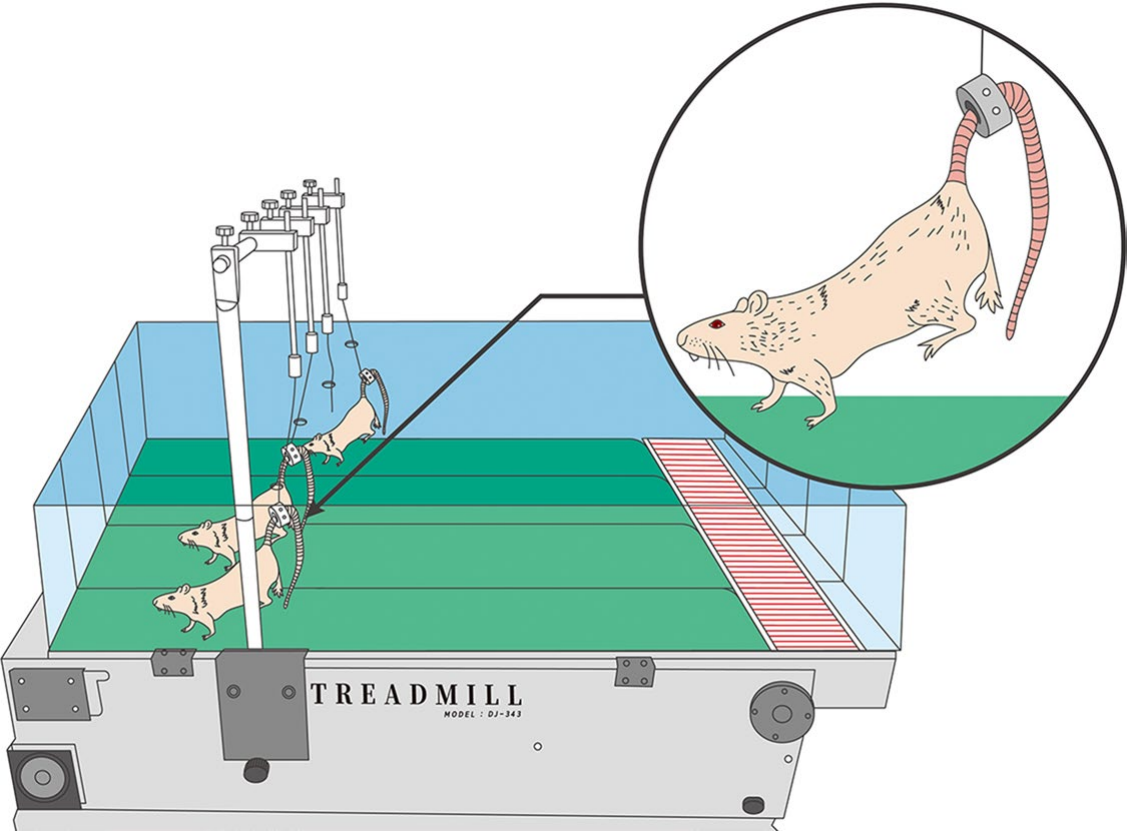
Illustration of treadmill walking exercise with hind paw suspension. In the high-intensity mechanical loading groups, the tails of the rats were fixed by gripping and lifting, forcing the rats to walk on the treadmill using only their front paws.

In the low-intensity mechanical loading groups, the rats were trained to walk on a treadmill (24 m/min, 0° inclination, 3 h/day, 5 days/week for 12 weeks). In the high-intensity mechanical loading groups, to increase the mechanical loading applied to the front paws, treadmill walking was performed with hind paw suspension by gripping and lifting the tails of the rats on the treadmill (Fig. 5). In the non-mechanical loading groups, rats were allowed only cage activity, and treadmill walking exercise was not conducted.

### Behavior test of mechanical allodynia

**Figure 6:**
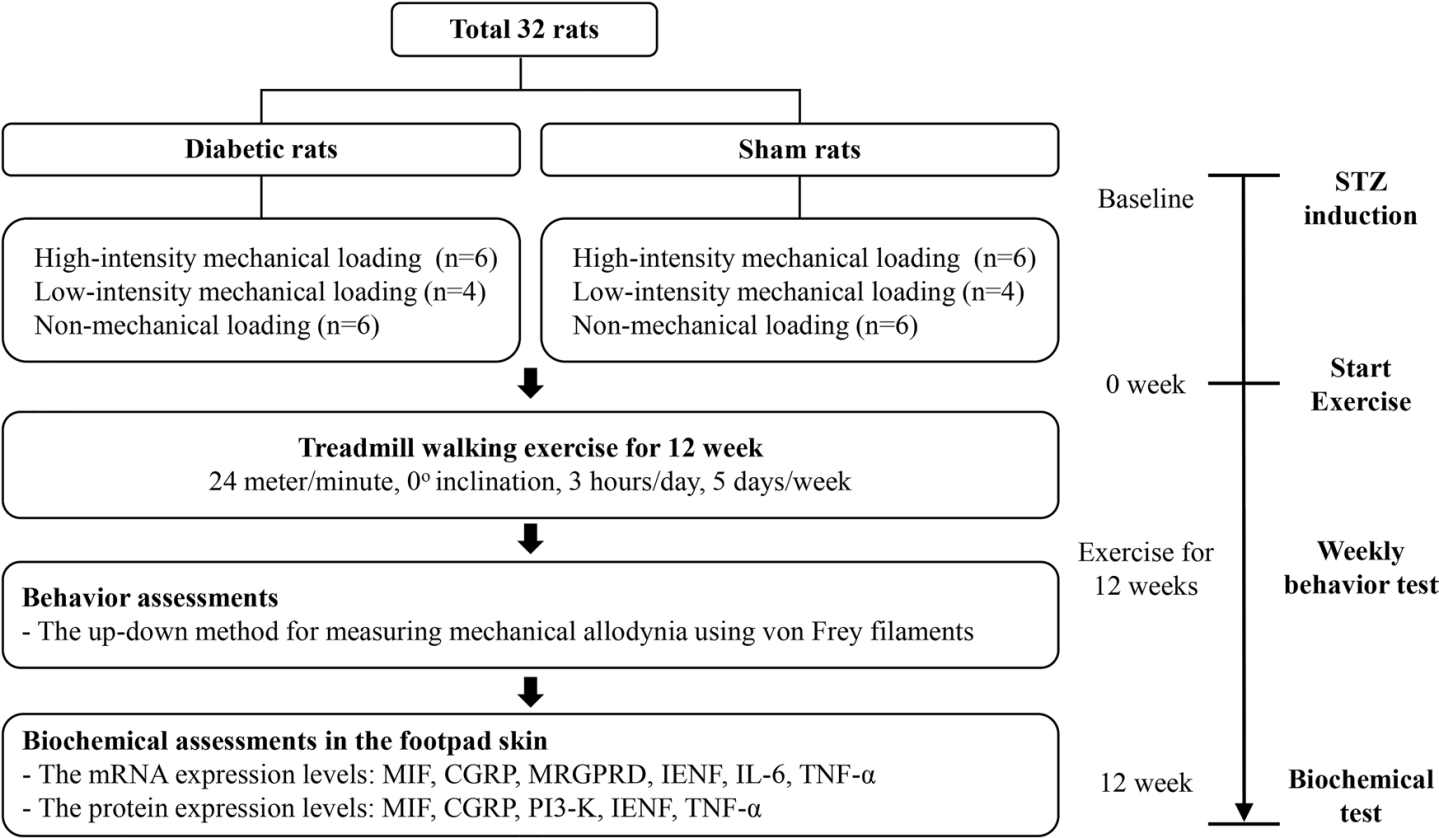
Study design and flow of the study. *MIF* macrophage migration inhibitory factor, *CGRP* calcitonin gene-related peptide, *MRGPRD* Mas-related G protein-coupled receptor D, *IENF* intraepidermal nerve fibers, *IL-6* interleukin-6, *TNF-α* tumor necrosis factor-alpha, *PI3-K* phosphoinositide 3-kinase, *STZ* streptozotocin.

Behavioral testing was performed after the induction of diabetes and every week for 12 weeks (Fig. 6). Mechanical allodynia was evaluated weekly by determining the rats’ 50% front paw withdrawal thresholds to von Frey monofilaments, as described by Chaplan et al.^45^. Briefly, rats were placed in individual enclosures in clean acrylic cages (19 × 25 × 14 cm) on a wire mesh grid located 70 cm above a table. After a 30 min habituation period in the test environment, rats were examined for mechanical allodynia. A set of von Frey monofilaments (0.4, 0.6, 1, 2, 4, 6, 8, and 15 g) was applied to the plantar surface of the front paw, beginning with the 2-g monofilament. Enough pressure was applied to cause the filament to buckle. Lifting, shaking, or licking of the front paw was considered a positive response and was followed by the use of the next weaker monofilament. Absence of paw withdrawal after 5 s indicated the use of the next higher weight monofilament. The process was repeated until the completion of evaluation according to the up and down method, and subsequently the 50% withdrawal threshold for each front paw was calculated using the equation provided by Chaplan et al.^45^. For consistency of the test, a single experienced investigator conducted all the behavioral evaluations.

### Tissue preparation

After the completion of the study, the rats were sacrificed for sample collection. Rats were anesthetized with 2.5% isoflurane and transcardially perfused with saline. Under aseptic conditions, the footpads of the front paws were isolated, excised, and immediately frozen in liquid nitrogen and stored at − 80 °C for analysis by real-time reverse transcription polymerase chain reaction (RT-PCR) and Western blotting.

### RNA extraction and real-time RT-PCR

RNA extraction and real-time RT-PCR were performed according to our previous study protocol^23^. Total RNA was extracted from the footpad skin tissues using TRIzol reagent (Invitrogen Life Technologies, Carlsbad, CA, USA). An equal amount of 10 µg total RNA was obtained from each sample and reverse-transcribed to cDNA with oligo (dT) 15 primers using M-MLV reverse transcriptase (Promega, Madison, WI, USA). The cDNAs were then subjected to real-time PCR using 2 × SYBR Green master mix kit (Roche Diagnostics, Indianapolis, IN, USA). Target gene-specific primer pairs were used (Table 1). Primer sequence specificity was confirmed by BLAST analysis for similar sequences against known sequence databases. PCR reaction mixtures were amplified and measured using SYBR Green fluorescence collected on a Light Cycler 480 system (Roche Diagnostics). Melting point analyses were carried out for each reaction to confirm single amplified products. The delta-delta Ct method was used to analyze gene expression levels normalized against β-actin expression. Experiments were performed in duplicate.

**Table 1:** Primer sequences for the rat genes.

	**Forward**	**Reverse**
MIF	5′-CCCAGAACCGCAACTACAGCAA-3′	5′-CGTTGGCTGCGTTCATGTCGTAAT-3′
MRGPRD	5′-CTGTCGAGTTTCCACAGGTTCC-3′	5′-TTGCGCAGAGGTACGGTTCC-3′
CGRP	5′-CTGTCGAGTTTCCACAGGTTCC-3′	5′-TTGCGCAGAGGTACGGTTCC-3′
IENF	5′-AGTGGCTCTCTGCAAAGCAG-3′	5′-GGCAGTAGAACGCAAGAAGA-3′
TNF-α	5′-CCCACGTCGTAGCAAACCACCA-3′	5′-CCATTGGCCAGGAGGGCGTTG-3′
IL-6	5′-CCTGGAGTTTGTGAAGAACAACT-3′	5′-GGAAGTTGGGGTAGGAAGGA-3′

*MIF* macrophage migration inhibitory factor, *MRGPRD* Mas-related G protein-coupled receptor D, *CGRP* calcitonin gene-related peptide, *IENF* intraepidermal nerve fibers, *TNF-α* tumor necrosis factor-alpha, *IL-6* interleukin-6.

### Protein isolation and Western blot analysis

Protein isolation and Western blot analysis protocols were based on our previous study^23^. Footpad tissues were lysed in a radioimmune precipitation assay buffer (Pierce, Rockford, IL, USA) with a protease inhibitor cocktail (Roche). The homogenates were centrifuged (10,000 RPM for 7 min at 4 °C), and supernatants were obtained. The protein content was quantified with bicinchoninic acid protein assay reagent (Pierce). Equal amounts of protein (20 µg) were separated by electrophoresis on 4–12% bis–tris gels and then transferred to a polyvinylidene difluoride membrane (Invitrogen). Membranes were stained with Ponceau S solution (Amresco, Fountain Parkway, Solon, OH, USA) to visualize protein bands and ensure equal protein loading and uniform transfer. After several washes, the membranes were incubated with 5% skim milk in Tris-buffered saline with Tween 20 for 1 h to block non-specific antigens. Membranes were incubated overnight at 4 °C with primary antibodies. Rabbit polyclonal antibody against macrophage MIF (Santa Cruz Biochemistry, Inc., Delaware, CA, USA) and mouse monoclonal antibodies against CGRP (Santa Cruz Biochemistry, Inc., Delaware, CA, USA), PI3-K (Santa Cruz Biochemistry, Inc., Delaware, CA, USA), IENF (Abcam, Cambridge, MA, USA), and β-actin (Santa Cruz Biochemistry, Inc., Delaware, CA, USA) were used in this study. The next day, after rinsing with Tris-buffered saline, the membranes were incubated with the corresponding conjugated anti-rabbit (1:5,000) or anti-mouse (1:5,000) horseradish peroxidase-coupled immunoglobulin G (Santa Cruz Biochemistry, Inc., Delaware, CA, USA). Immunoreactive proteins were visualized using an enhanced chemiluminescence system (Amersham Biosciences, Buckinghamshire, HP7 9NA, UK). ImageJ software (NIH, Bethesda, MD, USA) was used to measure the relative protein density, normalized against β-actin expression.

### Statistical analysis

All statistical analyses were performed by PASW Statistics 23.0 (SPSS, Chicago, USA). All data are presented as the mean ± standard deviation. For behavioral test data, two-way repeated measures analysis of variance (ANOVA) and the Bonferroni multiple-comparison post-hoc analysis were used for inter-group comparisons. To compare multiple groups, one-way ANOVA was used, followed by the Bonferroni multiple comparison post-hoc analysis. *P* < 0.05 was considered to indicate statistical significance.

All animal care handling and experimental procedures were carried out in accordance with a protocol approved by the Ethics Committee for Animal Experiments at Sungkyunkwan University, Kangbuk Samsung Hospital. This study has no commercial conflicts of interest.

## Supplementary information

Supplementary information

## Data Availability

Datasets used for the current study are not available publicly because that was not specified in the original ethical approval request. However, they can be made available in part from the corresponding authors upon reasonable request.
