# Supplementary material for: Excessive walking exercise precipitates diabetic neuropathic foot pain: hind paw suspension treadmill exercise experiment in a rat model
Source: Sci Rep. 2020 Jun 26;10:10498. doi: 10.1038/s41598-020-67601-6 (PMC7319951; doi:10.1038/s41598-020-67601-6)
Supplement: Supplementary file 1 — Supplementary information [file 41598_2020_67601_MOESM1_ESM.ppt]

## Slide 1
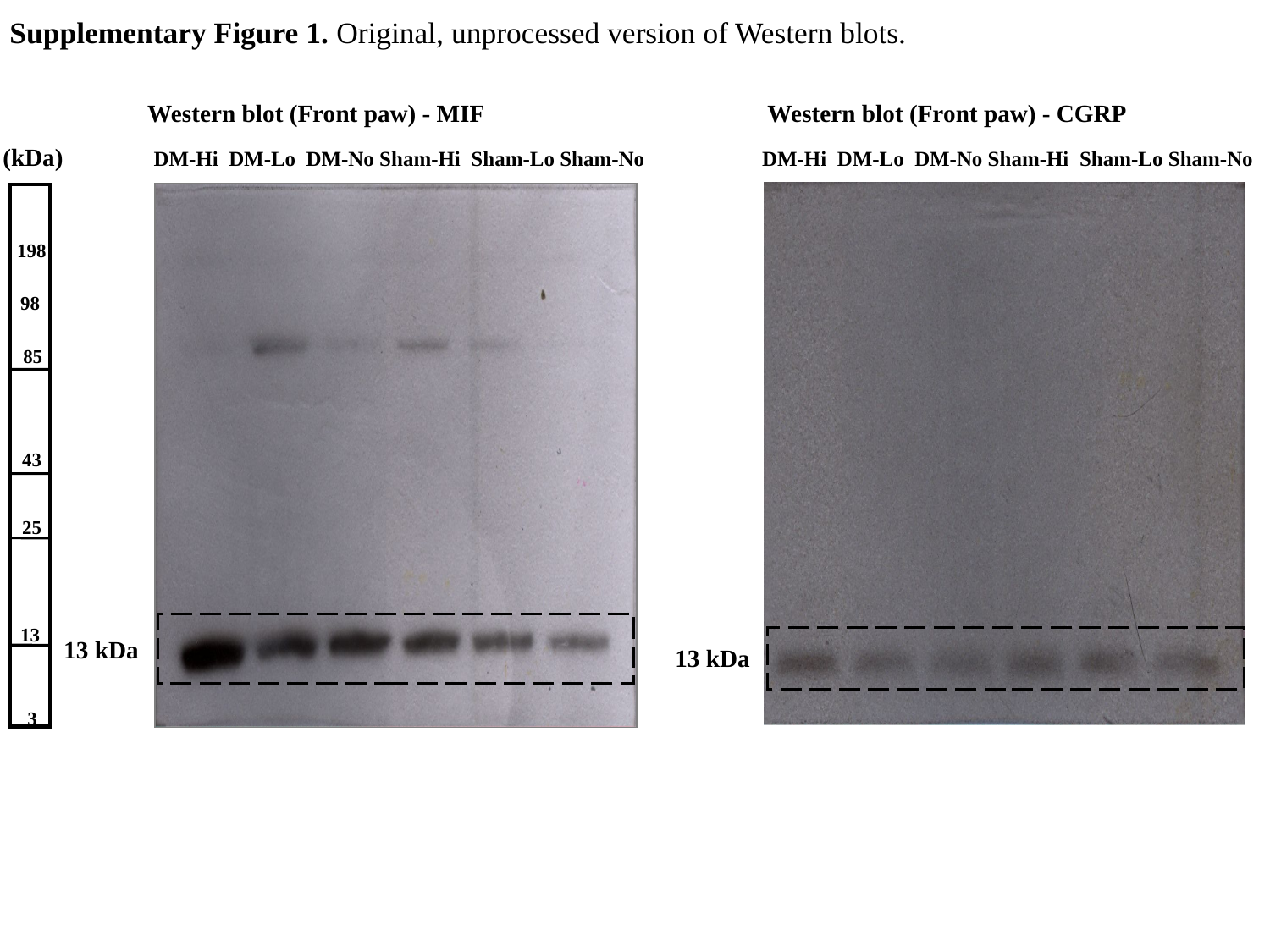

Supplementary Figure 1. Original, unprocessed version of Western blots.
Western blot (Front paw) - MIF
Western blot (Front paw) - CGRP
 DM-Hi DM-Lo DM-No Sham-Hi Sham-Lo Sham-No
 DM-Hi DM-Lo DM-No Sham-Hi Sham-Lo Sham-No
13 kDa
13 kDa
(kDa)
198
98
85
43
25
13
3

## Slide 2
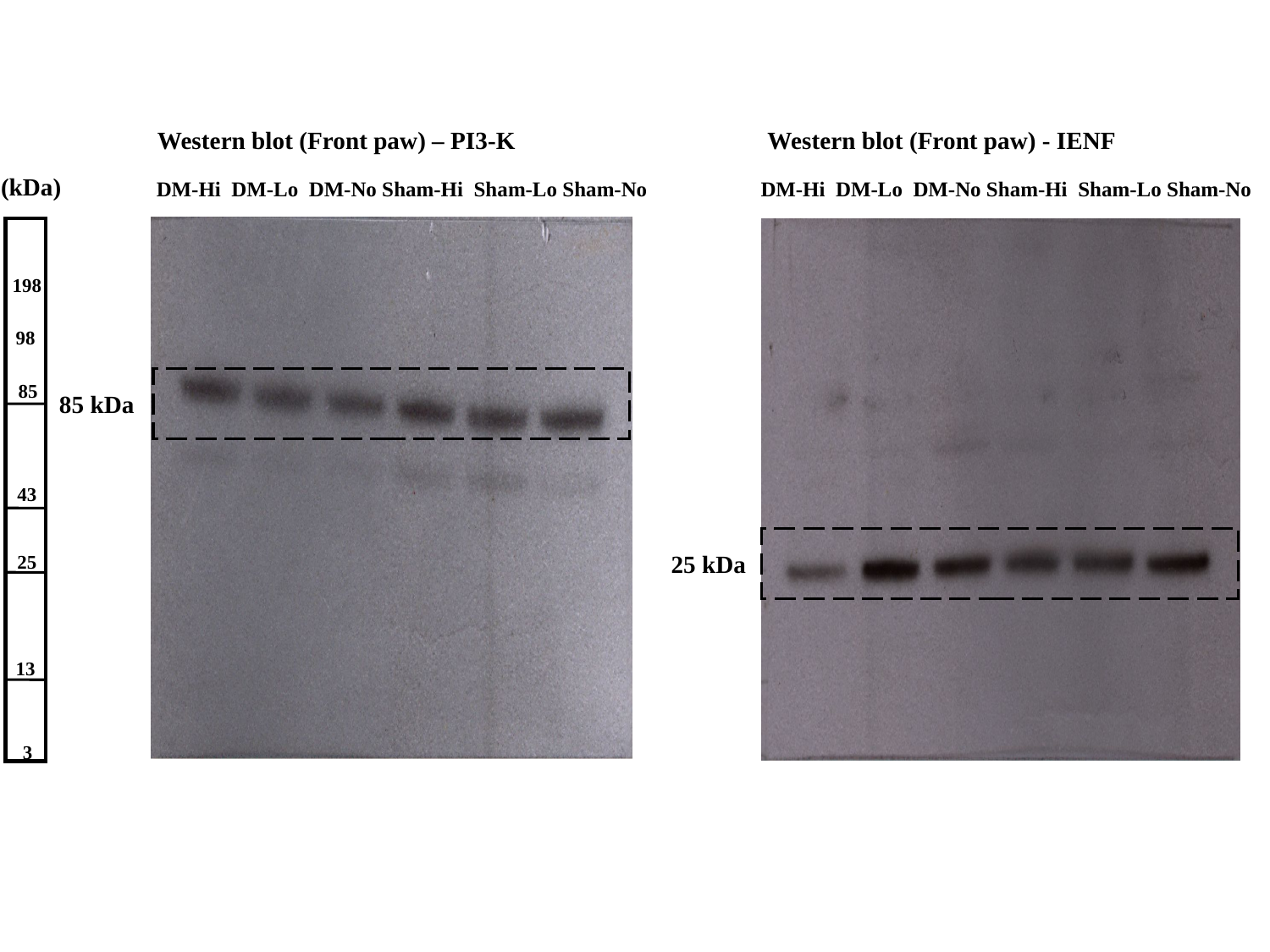

Western blot (Front paw) – PI3-K
Western blot (Front paw) - IENF
 DM-Hi DM-Lo DM-No Sham-Hi Sham-Lo Sham-No
 DM-Hi DM-Lo DM-No Sham-Hi Sham-Lo Sham-No
85 kDa
25 kDa
(kDa)
198
98
85
43
25
13
3

## Slide 3
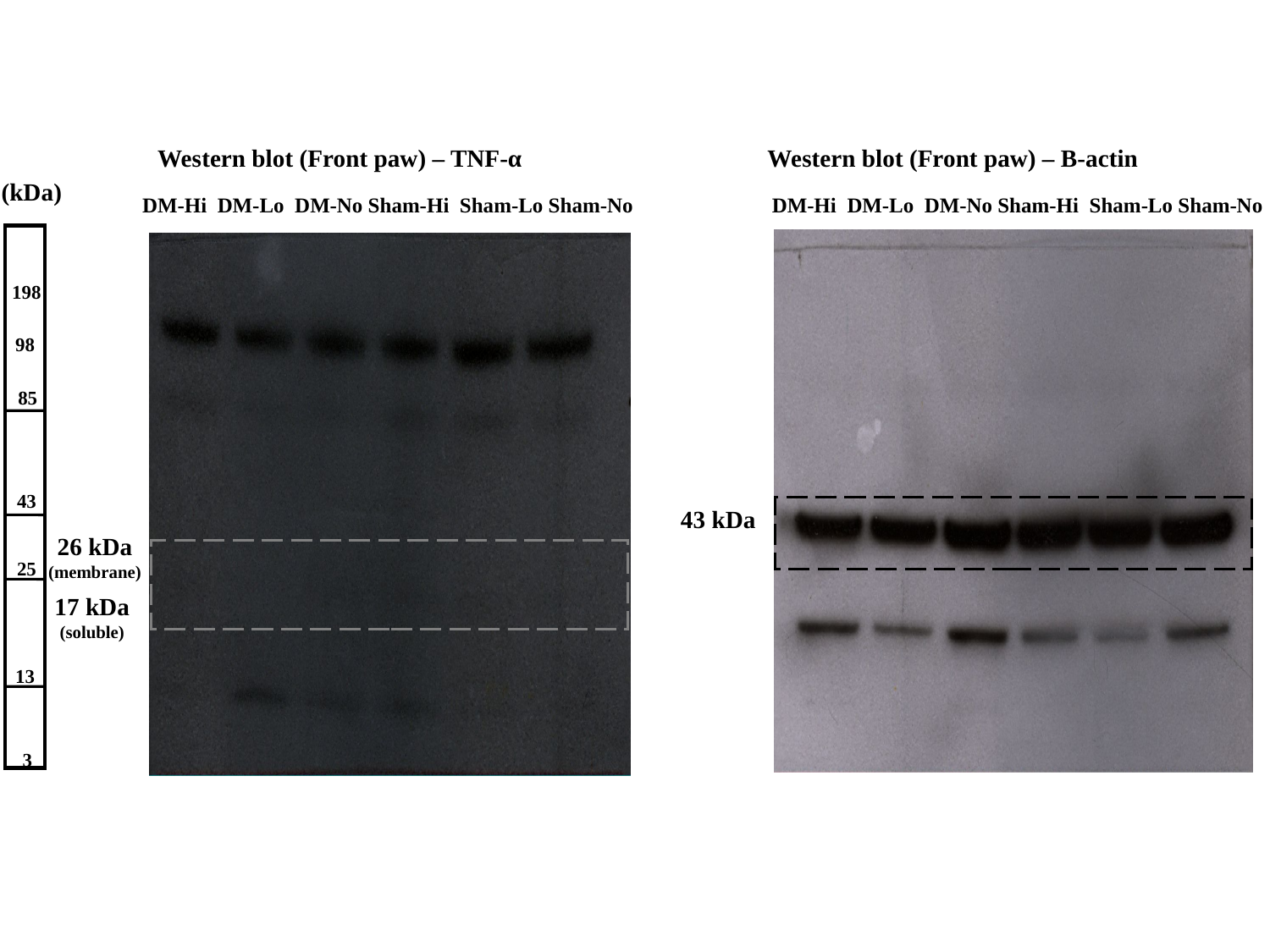

Western blot (Front paw) – B-actin
Western blot (Front paw) – TNF-α
 DM-Hi DM-Lo DM-No Sham-Hi Sham-Lo Sham-No
 DM-Hi DM-Lo DM-No Sham-Hi Sham-Lo Sham-No
43 kDa
26 kDa
(membrane)
17 kDa
(soluble)
(kDa)
198
98
85
43
25
13
3
